# Unsolved Questions in Salvage TIPSS: Practical Modalities for Placement, Alternative Therapeutics, and Long-Term Outcomes

**DOI:** 10.1155/2019/7956717

**Published:** 2019-04-01

**Authors:** Charlotte Bouzbib, Philippe Sultanik, Dominique Thabut, Marika Rudler

**Affiliations:** ^1^Intensive Care Unit, Hepatology Department, Pitié-Salpêtrière Charles Foix Hospital, Assistance Publique-Hôpitaux de Paris, Paris, France; ^2^Sorbonne University, Pierre et Marie Curie University, Paris, France

## Abstract

Salvage transjugular intrahepatic portosystemic shunt (TIPSS) has proven its efficacy to treat refractory variceal bleeding for patients with cirrhosis. However, this procedure is associated with very poor outcomes. As it is used as a last resort to treat a severe complication of cirrhosis, it seems essential to improve our practice, with the aim of optimizing management of those patients. Somehow, many questions are still unsolved: which stents should be used? Should a concomitant embolization be systematically considered? Is there any alternative therapeutic in case of recurrent bleeding despite TIPSS? What are the long-term outcomes on survival, liver transplantation, and hepatic encephalopathy after salvage TIPSS? Is this procedure futile in some patients? Is prognosis with salvage TIPSS nowadays as bad as earlier, despite the improvement of prophylaxis for variceal bleeding? The aim of this review is to summarize those data and to identify the lacking ones to guide further research on salvage TIPSS.

## 1. Introduction

Salvage transjugular intrahepatic portosystemic shunt (TIPSS) is a therapy that has proven its efficacy in patients with cirrhosis and refractory variceal bleeding. This latter diagnosis is made when patients present with variceal bleeding that does not respond to the combination of vasoactive drugs, endoscopic treatment, and antibiotherapy. Several studies have been published in this setting, showing that control of bleeding reaches 80 to 95% [1, 2]. The primary aim of this therapeutic is of course to stop bleeding in order to improve mortality and most of studies have focused on short-term survival.

There is currently no alternative therapeutics. Indeed, surgical shunts were considered before and were compared to TIPSS as a rescue therapy for variceal bleeding in 2 randomized controlled trials [3, 4]. Results seemed promising but had to be tempered by the fact that only bare stents were used in these studies, underestimating the benefits of TIPSS, and that surgical shunts need to be performed by experimented surgeons, available in rare centers nowadays, while TIPSS can technically be done in 90-100% of cases. Moreover, surgical shunts may prevent considering a later liver transplantation [5] and are associated with a higher morbidity and mortality than TIPSS [6, 7], explaining why TIPSS is the first-class therapeutic for refractory variceal bleeding.

Unfortunately, this procedure may cause severe complications, such as left-sided heart failure, right-sided heart failure for undiagnosed portopulmonary hypertension, hepatic encephalopathy (HE), and liver failure [6, 8]. The occurrence of such complications is significantly associated with death. Recent studies regarding nonurgent TIPSS suggest that polytetrafluoroethylene (PTFE)-covered stents improve patency and therefore lower the recurrence of complications of portal hypertension compared to bare stents [9, 10]. However, if ascites and variceal bleeding are the most severe complications of portal hypertension, patients may still develop heart failure, liver failure, and HE after TIPSS [6, 11, 12]. Due to its high incidence, HE is therefore the portal hypertension-related complication that needs improvement in terms of both prevention and treatment.

In this article, we will focus on unsolved questions regarding the particular situation of salvage TIPSS, which is rarely experienced in clinical practice. In the literature, 5-15% of patients with cirrhosis and acute variceal bleeding require salvage TIPSS [5, 13]. First, we will discuss which class of stents is recommended to treat a refractory bleeding. Second, we will address the alternative therapeutics in case of persistent bleeding despite TIPSS. Third, we will evaluate data on long-term benefits of salvage TIPSS. Fourth, we will review data on short-term prognosis in patients with very severe liver failure, to determine the risk of futility of this rescue strategy. Fifth, we will describe the prevalence of HE after salvage TIPSS and what treatment could be considered in this specific condition. Sixth, we will discuss the particular case of use of salvage TIPSS for ectopic varices. Seventh, we will discuss the possible effect of improved strategies for management of acute variceal bleeding on the use of salvage TIPSS in the last 5-10 years.

*(1) Which Class of Stents Should Be Used to Treat a Refractory Variceal Bleeding?* PTFE-covered stents have been used since the early 2000s in some centers. Nevertheless, all studies regarding salvage TIPSS were published before the use of covered stents. Specific data are therefore lacking. In nonurgent situations (refractory ascites, secondary prophylaxis for variceal bleeding), covered stents were found to reduce the risk of shunt obstruction without worsening the occurrence of HE after TIPSS [6, 9, 10]. In previously published studies, the rate of bare stents obstruction was poorly described but seemed to be as high as 50%, and even higher in patients presenting late rebleeding, i.e., after 7 days after initial bleeding (obstruction rate 87%) [3, 14]. Even if conducted in a small cohort of patients, the study by Sanyal and colleagues described a rate of 6-month stent stenosis of 50%, requiring dilation in most of cases [15].

Furthermore, the use of 10-mm stents has demonstrated a superiority compared to 8-mm stents to control refractory ascites in a retrospective cohort study [16] and in an early interrupted randomized controlled study [17]. However, benefits of small caliber covered stents to prevent a variceal rebleeding have been demonstrated [18]. Nevertheless, the effect of the stent diameter on post-TIPSS HE is controversial [6, 16–20]. In the context of salvage TIPSS, we should likely consider the use of 10-mm covered stents, which should be initially underdilated. Further dilation up to the nominal diameter should be decided on the basis of the final portosystemic pressure gradient [6, 21–25].

The benefits of variceal embolization concomitant with TIPSS placement are still controversial: some studies described that it may decrease the risk of recurrent bleeding after TIPSS [19–21], but other authors suggested that those results were not significant enough to justify its systematic appliance and that embolization should only be considered if there was recurrent bleeding [6, 26]. 

*(2) Is There any Alternative Therapeutic in Case of Rebleeding after Salvage TIPSS?* Most of patients who present rebleeding after salvage TIPSS will die [27]. Data on other therapeutics are lacking in the literature. In most of cases, rebleeding after TIPSS is related to TIPSS obstruction: it is therefore mandatory to check the patency of TIPSS in such situations. The gold standard is a direct opacification of the stent and a subsequent thrombectomy may be attempted if indicated. Moreover, a variceal embolization should be considered if possible [26]. Finally, in patients with persistent bleeding and high MELD score, liver transplantation is the best option and a balloon tamponade or a self-expending metal esophageal stent could be used as a bridge to surgery [6]. Usually, patients requiring a salvage TIPSS have decompensated cirrhosis with high Child-Pugh and MELD scores [14, 15, 27, 28], except for very rare instances such as splanchnic vein thrombosis, or ectopic varices, or both. In these latter patients, liver transplantation could be prioritized based on a MELD score exception. This would require a thorough assessment by a liver transplant expert mandated for each particular case [6, 29]. If accepted, it would then result in a very short waiting time on the transplant waiting list. 

*(3) Do We Have Long-Term Data on Survival and Need for Liver Transplantation?* As salvage TIPSS is required to stop bleeding in unresponsive patients, the assessment of the performance of this therapeutic focused on short-term survival (7 or 42 days). Most of studies have provided data on survival with a median follow-up of less than 1 year. Nevertheless, in the study conducted by Sanyal and colleagues, after a median follow-up of 920 days, 46% of the original cohort was alive [15]. In contrast, the study conducted by Henderson showed a five-year survival rate of 61% after salvage TIPSS placement in 67 patients, exclusively with Child-Pugh A or B cirrhosis, which may explain this high rate of survival [30]. In a French study, the actuarial survival rate following salvage TIPSS was 51.7%, 40.2%, and 40.2% at 1, 3, and 5 years, respectively [31]. Data on liver transplantation are only available in 3 studies: 8/68 (12%) patients underwent liver transplantation in the study conducted by Jalan and colleagues [1], while it was the case of 6/58 (10%) patients in the study of Azoulay et al. [31] and 3/18 (17%) in the study of Sanyal et al. [15]. Neither short-term nor long-term transplant-free survival was described. 

*(4) Is Salvage TIPSS a Futile Procedure for Patients in the Most Severe Condition?* Although data are limited, mortality is high in patients in whom salvage TIPSS failed to control bleeding or those with multiorgan failure. Mortality is strongly associated with hyperbilirubinaemia [32], renal failure [33, 34], hyponatremia [1], sepsis, use of catecholamines, and a high APACHE II score [1, 2, 15, 32–36]. Severity of cirrhosis at the time of bleeding is often associated with mortality [1, 37] and liver transplantation after TIPSS has to be considered in this situation. Unfortunately, one cannot identify patients for which TIPSS placement will be futile. A few years ago, we reported a series of cirrhotic patients with refractory variceal bleeding and Child-Pugh C14 or 15 scores. In-hospital transplant-free mortality was 100%. After the implementation of the MELD score for allocation of liver grafts in France in 2007, we successfully performed a rapid liver transplantation in 5 consecutive good candidates after salvage TIPSS placement. One-year outcome was excellent in this small cohort (100% survival) [38]. Based on this limited amount of data, we usually consider that salvage TIPSS is futile in patients with Child-Pugh C14 or 15 cirrhosis [39] who will not be candidates for liver transplantation. As no recommendation can be clearly given, an expert's advice is mandatory for each case.

*(5) What Proportion of Patients Will Develop HE after TIPSS? Are There Pre-TIPSS Risk Factors of HE and Are There Specific Treatments to Improve this Condition?* As already stated, the main goal of salvage TIPSS is to save patients from refractory bleeding. Therefore, neurological complications after TIPSS have not been extensively described. Among previously published studies, only few evaluated the prevalence of HE in patients that needed salvage TIPSS [1, 14, 15, 30, 32, 40]: it ranged from 20% to 90%, reflecting the variability of severity of patients and more obviously the variability of clinical evaluation. There is also an issue regarding the evolution of HE after salvage TIPSS, as no study clearly described the predictive factors of worsening or improving HE after TIPSS. However, in case of HE refractory to conventional therapy after TIPSS, benefits of specific treatments as TIPSS reduction have been proven [41–44]. Somehow, those benefits should be balanced with the risk of other portal hypertension-related complications, especially with the potential risk of rebleeding [42]. This aspect has still to be studied in the setting of refractory bleeding.

*(6) Should Salvage TIPSS Be Considered to Treat Ectopic Variceal Bleeding?* In some cases, treatment of acute variceal bleeding in agreement with guidelines is not possible, particularly because of impossibility of endoscopic treatment. This is the case for ectopic varices, for which bleeding is rare: it concerns about 2-5% of bleeding episodes in patients with cirrhosis. Treatment is particularly difficult because of a frequent delayed diagnosis and a complexity to reach varices and to treat them, both during the acute bleeding episode and in secondary prophylaxis. Thus, we may have recourse to salvage TIPSS in this setting. Indeed, TIPSS has been described in many case reports and in some small series as a good therapeutic for ectopic varices [26, 45–47]: it allows control of bleeding in most of cases (90-100%), with a poor rate of recurrent bleeding (15-30%) and complications, provided the small size of those series. Whether concomitant embolization of varices should be performed is still matter of debate, as this may increase length of procedure, costs, and irradiation [48], even if it seems to prevent rebleeding [16, 18, 19, 30].

*(7) Has the Early-TIPSS Policy Had an Impact on the Recourse to Salvage TIPSS? *Since salvage TIPSS is indicated for refractory variceal bleeding, one could argue that a number of these bleeding episodes could have been avoided if a better prophylaxis of (re-)bleeding had been implemented. Variceal bleeding prophylaxis improved considerably over the past years, partly due to the early-TIPSS policy [49–52]. Since Baveno V in 2010 [53, 54], preemptive TIPSS is recommended after a variceal bleeding in patients with cirrhosis and high risk of rebleeding, defined by a Child-Pugh B score and active bleeding at endoscopy or Child-Pugh C score lower than 14. This policy contributed to the decrease in the recurrence of bleeding in severe patients and therefore could have drastically reduced the use of salvage TIPSS. Further studies are needed to corroborate this view.

## 2. Conclusion

There are no recent data in the literature on salvage TIPSS. Further studies are warranted to assess the current outcomes after salvage TIPSS, especially with the systematic use of PTFE-covered stents. Moreover, new strategies have been developed for the last years to improve the prophylaxis for variceal bleeding, such as the early-TIPSS policy. Whether this policy reduces the recourse to salvage TIPSS has to be investigated. Last, data on long-term survival, HE and the need for liver transplantation after TIPSS are unfortunately lacking.

## Abbreviations

TIPSS: Transjugular intrahepatic portosystemic shunt
HE: Hepatic encephalopathy
PTFE: Polytetrafluoroethylene
MELD: Model for End-Stage Liver Disease.
